# Discovery and functional characterization of two diterpene synthases for sclareol biosynthesis in *Salvia sclarea* (L.) and their relevance for perfume manufacture

**DOI:** 10.1186/1471-2229-12-119

**Published:** 2012-07-26

**Authors:** Anne Caniard, Philipp Zerbe, Sylvain Legrand, Allison Cohade, Nadine Valot, Jean-Louis Magnard, Jörg Bohlmann, Laurent Legendre

**Affiliations:** 1 Michael Smith Laboratories, University of British Columbia, 301-2185 East Mall, Vancouver, BC, V6T 1Z4, Canada; 2 Université Lille Nord de France, Lille, F-59000, France; 3 Université Lille1, Villeneuve d’Ascq, F-59655, France; 4 Stress Abiotiques et Différenciation des Végétaux Cultivés (SADV), UMR INRA 1281, Bâtiment SN2, Villeneuve d'Ascq, F-59655, France; 5 Université de Lyon, Saint-Etienne, F-42023, France; 6 Université de Saint-Etienne, Jean Monnet, Saint-Etienne, F-42000, France; 7 Laboratoire BVpam, EA3061, 23 rue du Dr Paul Michelon, Saint-Etienne, F-42000, France; 8 Université de Lyon, Lyon, F-69622, France; 9 Université Lyon 1, Villeurbanne, France; 10 CNRS, UMR5557, Ecologie Microbienne, Villeurbanne, France

**Keywords:** Diterpene, Sage, *Salvia sclarea*, Sclareol, Terpene synthase

## Abstract

**Background:**

Sclareol is a diterpene natural product of high value for the fragrance industry. Its labdane carbon skeleton and its two hydroxyl groups also make it a valued starting material for semisynthesis of numerous commercial substances, including production of Ambrox® and related ambergris substitutes used in the formulation of high end perfumes. Most of the commercially-produced sclareol is derived from cultivated clary sage (*Salvia sclarea*) and extraction of the plant material. In clary sage, sclareol mainly accumulates in essential oil-producing trichomes that densely cover flower calices. Manool also is a minor diterpene of this species and the main diterpene of related *Salvia* species.

**Results:**

Based on previous general knowledge of diterpene biosynthesis in angiosperms, and based on mining of our recently published transcriptome database obtained by deep 454-sequencing of cDNA from clary sage calices, we cloned and functionally characterized two new diterpene synthase (diTPS) enzymes for the complete biosynthesis of sclareol in clary sage. A class II diTPS (*Ss*LPPS) produced labda-13-en-8-ol diphosphate as major product from geranylgeranyl diphosphate (GGPP) with some minor quantities of its non-hydroxylated analogue, *(9 S, 10 S)*-copalyl diphosphate. A class I diTPS (*Ss*SS) then transformed these intermediates into sclareol and manool, respectively. The production of sclareol was reconstructed *in vitro* by combining the two recombinant diTPS enzymes with the GGPP starting substrate and *in vivo* by co-expression of the two proteins in yeast (*Saccharomyces cerevisiae*). Tobacco-based transient expression assays of green fluorescent protein-fusion constructs revealed that both enzymes possess an N-terminal signal sequence that actively targets *Ss*LPPS and *Ss*SS to the chloroplast, a major site of GGPP and diterpene production in plants.

**Conclusions:**

*Ss*LPPS and *Ss*SS are two monofunctional diTPSs which, together, produce the diterpenoid specialized metabolite sclareol in a two-step process. They represent two of the first characterized hydroxylating diTPSs in angiosperms and generate the dihydroxylated labdane sclareol without requirement for additional enzymatic oxidation by activities such as cytochrome P450 monoxygenases. Yeast-based production of sclareol by co-expresssion of *Ss*LPPS and *Ss*SS was efficient enough to warrant the development and use of such technology for the biotechnological production of scareol and other oxygenated diterpenes.

## Background

Diterpenoids constitute a large class of chemically diverse metabolites that is widely distributed throughout the plant kingdom with more than 12,000 known compounds, the majority of which derives from bicyclic ‘labdane-related’ diterpene intermediates [1]. These include the gibberellin phytohormones as part of general (i.e. primary) plant metabolism with essential roles in plant growth and development [2-4] and a plethora of specialized (i.e. secondary) metabolites with essential functions in ecological interactions of plants with other organisms, including attraction of pollinators or defense against pests or pathogens [5-8]. Because of their various biological activities in humans, diterpenoids of plant origins are of substantial economical relevance as bioproducts for a variety of applications, for example as pharmaceuticals or as fragrance components [9,10].

**Figure 1:**
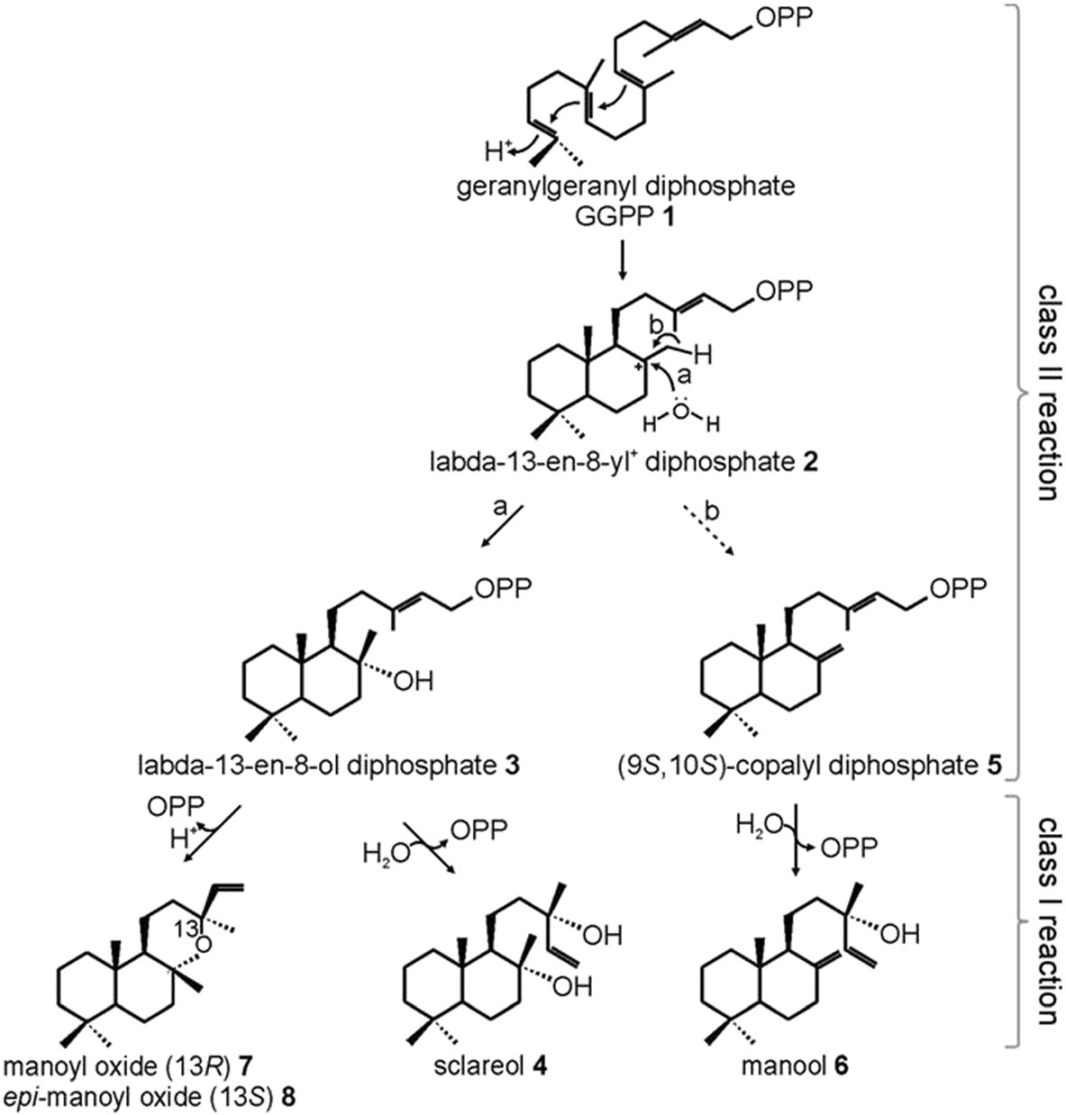
**Proposed biosynthetic pathway of sclareol and related diterpenes in *Salvia sclarea.*** The suggested biosynthetic pathway of sclareol 4 as the predominant diterpene in *S. sclarea* and other minor constituents detected *in planta*, such as manool 6 as well as manoyl oxide 7 and 13-*epi*-manoyl oxide 8, requires the activity of at least two monofunctional diTPSs. A class II enzyme catalyzes the protonation-initiated cyclization of (*E*,*E*,*E*)-geranylgeranyl diphosphate (GGPP) 1 to form labda-13-en-8- ol diphosphate (LPP)3 or (9 *S*,10 *S*)-copalyl diphosphate (CPP) 5 (i.e., CPP of normal or (+)-stereochemistry) via a labda-13-en-8-yl diphosphate 2 carbocation. Catalyzed by class I diTPS activity, ionization of the diphosphate ester of LPP 3 or CPP 5 results in the formation of sclareol 4 and manool 6, respectively. In addition, manoyl oxide 7 and 13-*epi*-manoyl oxide 8 may occur as a product of this biosynthetic pathway.

Sclareol (Figure 1) is a labdane-type diterpene alcohol, which has been reported in four plant species of four different families: *Salvia sclarea* (*Lamiaceae*) [11], *Cistus creticus* (*Cistaceae*) [12], *Nicotiana glutinosa* (*Solanaceae*) [13] and *Cleome spinosa* (*Brassicaceae*) [14]. Although sclareol has been suggested to possess anti-bacterial, anti-fungal and growth regulating activities, its function *in planta* is unclear [12,15-19]. A major use of sclareol is in the fragrance industry. Sclareol is the most common starting material for the synthesis of Ambrox® [20], which serves as a valuable and sustainable substitute for ambergris [21], a waxy substance secreted by sperm whales. Ambergris has historically been appreciated for its musky and sweet earthy odor and has been used for many years as a fixative in high-end perfumes. However, its origin from an endangered and protected animal species made the use of ambergris in the fragrance industry controversial.

Clary sage (*Salvia sclarea*) is the plant species predominantly used for production and isolation of sclareol. It is a native species of the Mediterranean basin, Southern Europe and Iran, and is commercially grown mostly in Europe (France, Hungary, Bulgaria) and North America for its essential oils [22]. Despite successful cultivation of clary sage, annual production and availability of sclareol varies substantially due to uncertain environmental factors. The development of a cost-efficient and scalable enzymatic production platform would improve the reliability of sclareol production. However, the genes and enzymes responsible for sclareol biosynthesis have not been described.

In general, the biosynthesis of bicyclic labdane-type diterpenes proceeds via stepwise ionization and cycloisomerization of (*E,E,E*)-geranylgeranyl diphosphate (GGPP). In angiosperms, such a reaction cascade requires the consecutive activity of two monofunctional diterpene synthases (diTPSs). A class II diTPS catalyzes the protonation-dependent cyclization of GGPP to form a bicyclic diphosphate intermediate of variable stereochemistry and hydroxylation. Subsequently, class I diTPSs facilitate the ionization of the diphosphate group and often additional cyclization and rearrangement reactions [23-25]. In angiosperms and gymnosperms, functional modification of labdane and labdane-related diterpenoids involves primarily addition of hydroxy groups, which can be mediated by diTPSs [26-28] or through the activity of cytochrome P450-dependent monooxygenases (P450s) [28-35].

Based on the established general patterns of diterpene biosynthesis in angiosperms, we propose that biosynthesis of sclareol in clary sage may involve the activity of two monofunctional diTPSs (Figure 1). In a plausible sequence of diTPS activities, a class II diTPS may first catalyzes the bicyclization of GGPP (1) and water capture at C-8 to afford labda-13-en-8-ol diphosphate (LPP, 3), similar to the function of copal-8-ol diphosphate synthase (*Cc*CLS) from *C. creticus*[25]. Subsequently, a class I diTPS may convert LPP through cleavage of the diphosphate group and may also catalyze the additional hydroxylation at C-13 to form sclareol (4). A recent patent [36] reported on two genes [GenBank: AET21247, GenBank: AET21246] coding for similar enzymatic activities in *S. sclarea*, however lacking a complete description of the enzyme activities. Hydroxylation reactions in the class I actives site of diTPSs have been reported for bifunctional class I/II diTPS outside of the angiosperms, namely copalyl diphosphate / kaurene synthases (CPS/KS) from the non-vascular plants *Physcomitrella patens* and *Jungermannia subulata*[28,37], labda-7,13-dien-15-ol synthase from the lycophyte *Sellaginella moellendorffii*[27], and levopimaradiene / abietadiene synthase from *Picea abies* (*Pa*LAS) [26].

Using previously established transcriptome sequence resources for clary sage calyces [38], we describe here the isolation of full-length (FL)-cDNAs of a class II diTPS (*Ss*LPPS) and two class I diTPSs (*Ss*SS and *Ss*diTPS3). We show that the enzymes encoded by *SsLPPS* and *SsSS* catalyze the direct formation of sclareol without the requirement of a P450-mediated hydroxylation. We demonstrate the subcellular localization of both sclareol-biosynthetic diTPSs in plastids. Initial efforts of engineering of sclareol biosynthesis in yeast established promising leads for the future development of microbial production systems for sclareol using plant enyzmes.

## Results

### Transcriptome mining and discovery of SsLPPS, SsSS and SsdiTPS3 cDNAs

We hypothesized that sclareol is synthesized from GGPP through a two-step mechanism involving a pair of class II and class I monofunctional diTPS (Figure 1). Given the high abundance of sclareol in metabolite extracts of clary sage calyces, this tissue was subjected to 454 pyrosequencing and revealed six different diTPS candidate sequences [38]. Additional data mining of the 454-sequences allowed the retrieval of two additional sequences presenting homologies with known diTPSs. Full length sequencing of the cDNAs of these eight candidate sequences recovered by 5’- and 3’-RACE (Additional file 1: Figure S1) revealed that they were independent parts of three separate diTPS genes, a class II diTPS (*Ss*LPPS) containing the characteristic DxDD motif, and class I diTPSs (*Ss*SS and *Ss*diTPS3) carrying the conserved DDxxD and NSE/DTE functional motifs.

### Phylogenetic analysis

**Figure 2:**
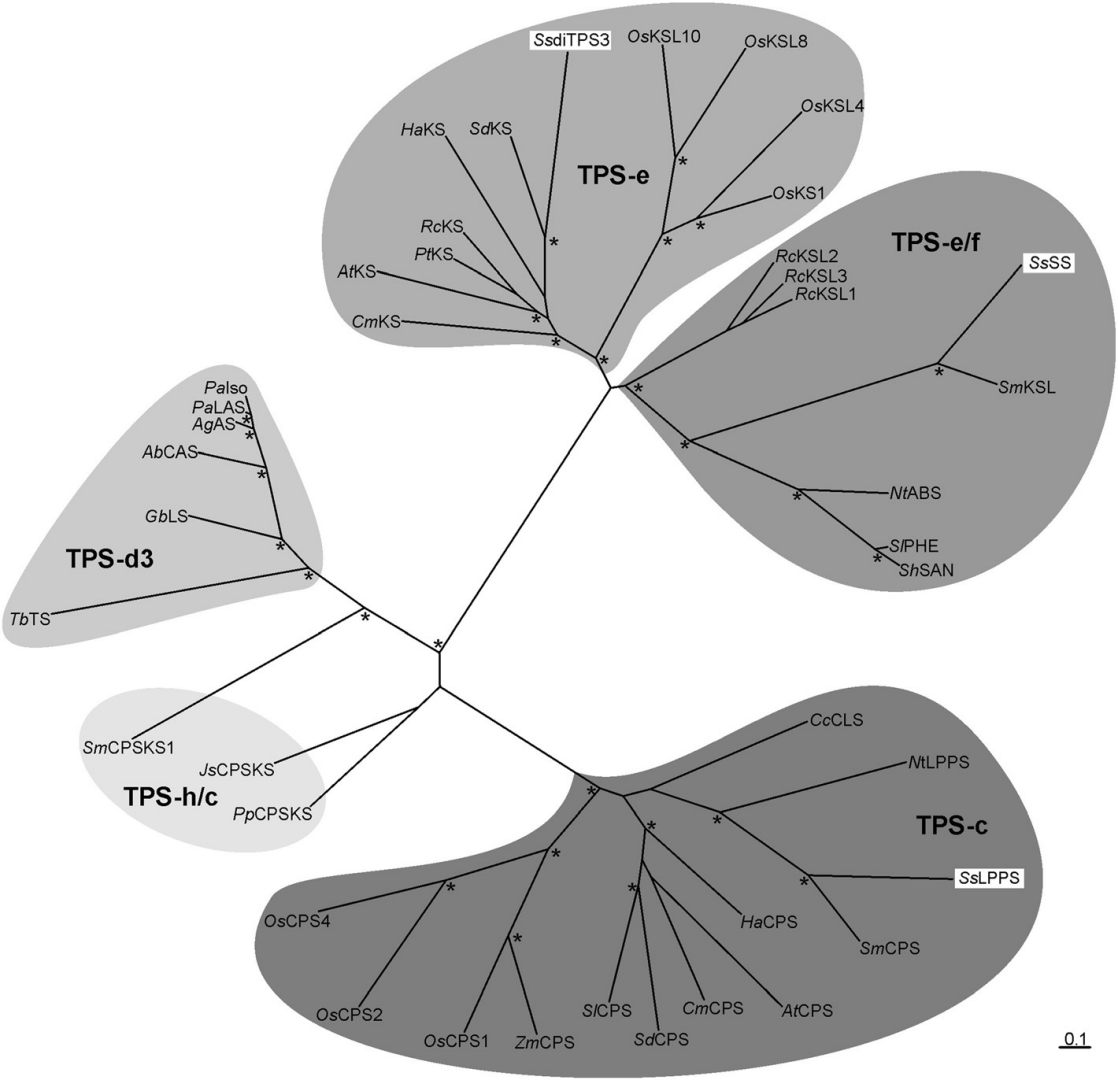
**Figure 2 – Phylogenetic analysis of Salvia sclarea diterpene synthases.** The phylogenetic tree was generated based on multiple amino acid sequence alignments (DIALIGN-TX), phylogenetic analysis (PhyML, four rate substitution categories, LG substitution model, BIONJ starting tree, 100 bootstrap repetitions, rooted with the outgroup copalyl diphosphate synthase/kaurene synthase from the moss *Physcomitrella patens* PpCPS/KS [BAF61135]), and visualization in treeview. Asterisks indicate nodes supported by > 90% bootstrap values. Protein abbreviations [NCBI GenBank accession no. ]: JsCPS/KS, *Jungermannia subulata ent*-copalyl diphosphate/*ent*-kaurene synthase [BAJ39816]; *Sm*CPS/KS1, *Selaginella moellendorfii* labda-7,13E-dien-15-olsynthase [AEK75338]; *Tb*TS, *Taxus brevifolia* taxadiene synthase [AAC49310]; *Gb*LS, *Ginkgo biloba* levopimaradiene synthase [AAL09965]; *Ab*CAS, *Abies balsamea cis*-abienol synthase [JN254808]; *Ag*AS, *Abies grandis* abietadiene synthase [AAK83563]; *Pa*LAS, *Picea abies* levopimaradiene/abietadiene synthase [AAS47691]; *Pa*Iso, *Picea abies* isopimaradiene synthase [AAS47690]; *Cm*KS, *Cucurbita maxima* kaurene synthase [AAB39482]; *At*KS, *Arabidopsis thaliana ent*-kaurene synthase [AF034774]; *Pt*KS, *Populus trichocarpa* kaurene synthase [XM_002311250]; *Rc*KS, *Ricinus communis* kaurene synthase-like [XP_002533694]; *Ha*KS, *Helianthus annuus* kaurene synthase [CBL42917]; *Sd*KS, *Scoparia dulcis* kaurene synthase-like [AEF33360]; *Os*KS1, *Oryza sativa ent*-kaurene synthase [AY347876]; *Os*KSL4, *O. sativa syn*-pimaradiene synthase [AY616862]; *Os*KSL8, *O. sativa syn*-stemarene synthase [AB118056]; *Os*KSL10, *O. sativa ent*-pimaradiene synthase [DQ823355]; *Rc*KSL1, R. communis kaurene synthase-like [XM_002525795]; *Rc*KSL2, *R. communis* kaurene synthase-like [XM_002525790]; *Rc*KSL3, *R. communis* kaurene synthase-like [XM_002525796]; *Sm*KSL, *Salvia miltiorhizza* kaurene synthase-like [EF635966]; *Nt*KSL, *N. tabacum* kaurene synthase-like [CCD33019]; *Sl*PHE, *Solanum lycopersicum* phellandrene synthase [FJ797957]; *Sh*SBS, *Solanum habrochaites* bergamotene/santalene synthase [FJ194970]; *Cc*CLS, *Cistus creticus* copal-8-ol synthase [DJ93862]; *Nt*CPSL, *Nicotiana tabacum* 8-hydroxy copalyl diphosphate synthase [CCD33018]; *Sm*CPSL, *S. miltiorhizza* copalyl diphosphate synthase-like [EU003997]; *Ha*CPS, *H. annuus* copalyl diphosphate synthase [CBL42915]; *At*CPSL, *A. thaliana ent*-copalyl diphosphate synthase [AAA53632]; *Cm*CPS, *C. maxima* copalyl diphosphate synthase [AF049905]; *Sd*CPSL, *S. dulcis* copalyl diphosphate synthase-like [AB046689]; *Sl*CPSL, *S.lycopersicum* copalyl diphosphate synthase-like [AB015075]; *Zm*CPSL, *Zea mays ent*-copalyl diphosphate synthase [AY562491]; *Os*CPS1, *O. sativa ent*-copalyl diphosphate synthase [Q6ET36]; *Os*CPS2, *O. sativa ent*-copalyl diphosphate synthase [Q6Z5I0]; *Os*CPS4, *O. sativa syn*-copalyl diphosphate synthase [Q6E7D7].

Phylogenetic comparison of the translated FL-cDNA sequences confirmed the assignment of *Ss*LPPS to the TPS-c subfamily of angiosperm class II diTPSs [39,40] (Figure 2). Specifically, *Ss*LPPS is closer related to class II diTPSs that are involved in specialized metabolism such as (9 *S*,10 *S*)-CPP (i.e. CPP of normal stereochemistry) synthase from *Salvia miltiorhizza*[24] and copal-8-ol diphosphate synthase from *C. creticus* (C*c*CLS) [25]. *Ss*SS and *Ss*diTPS3 can be assigned to the TPS-e/f and TPS-e families respectively, which contain KS-like enzymes involved in general or specialized diterpene metabolism [39].

Interestingly, *Ss*SS lacks the internal γ-domain, which is characteristic of the archetype three-domain structure of plant diTPSs [41-44]. While the γ-domain is essential during class II diTPS catalysis, the active site of a monofunctional class I diTPS is located in the α-domain. *Ss*SS is closely related to a recently reported class I diTPS from *S. miltiorhizza* that produces miltiradiene and exhibits a similar loss of the γ-domain [24,43]. Together, the phylogenetic relation and domain structure suggested that *Ss*SS encodes a monofunctional class I diTPS involved in specialized metabolism. In contrast, *Ss*diTPS3 exhibits the common αβγ architecture and shares only 23.5% identity with *Ss*SS. Its closer relation to *ent*-kaurene synthases within the TPS-e subfamily may suggest a function in general rather than specialized metabolism.

In summary, *Ss*LPPS, *Ss*SS and *Ss*diTPS3 represent the three different subfamilies of angiosperm diTPSs involved in the biosynthesis of labdane-type diterpenoids (Figure 2).

### Functional characterization of *S. sclarea* diTPSs and discovery of sclareol synthase

**Figure 3:**
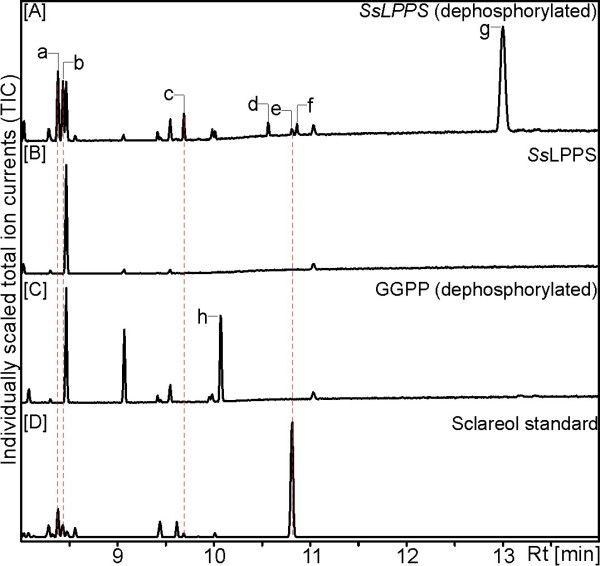
**GC-MS analysis of *Ss*LPPS reaction products.** Shown are total ion chromatograms (TIC) of [**A**] reaction products obtained from *in vitro* assays with Ni^2+^-affinity-purified *Ss*LPPS using GGPP **1** as a substrate and subsequent dephosphorylation, [**B**] *in vitro* assay products without dephosphorylation, [**C**] dephosphorylated GGPP, and [**D**] authentic sclareol standard (Sigma). GC-MS analysis was performed on an Agilent HP5ms column with electronic impact ionization at 70 eV. Results were confirmed with three independent experiments. Identification of reaction products was achieved by comparison to authentic standards or reference mass spectra from the National Institute of Standards and Technology MS library searches (Wiley W9N08L): peak a, *epi*-manoyl oxide **8**; peak b, manoyl oxide **7**; peak c, putative 13(16),-14-labdien-8-ol; peak d, putative copalol; peak e, sclareol **4**; peak f, unknown compound; peak g, labda-13-en-8,15-diol; peak h, geranylgeraniol.

**Figure 4:**
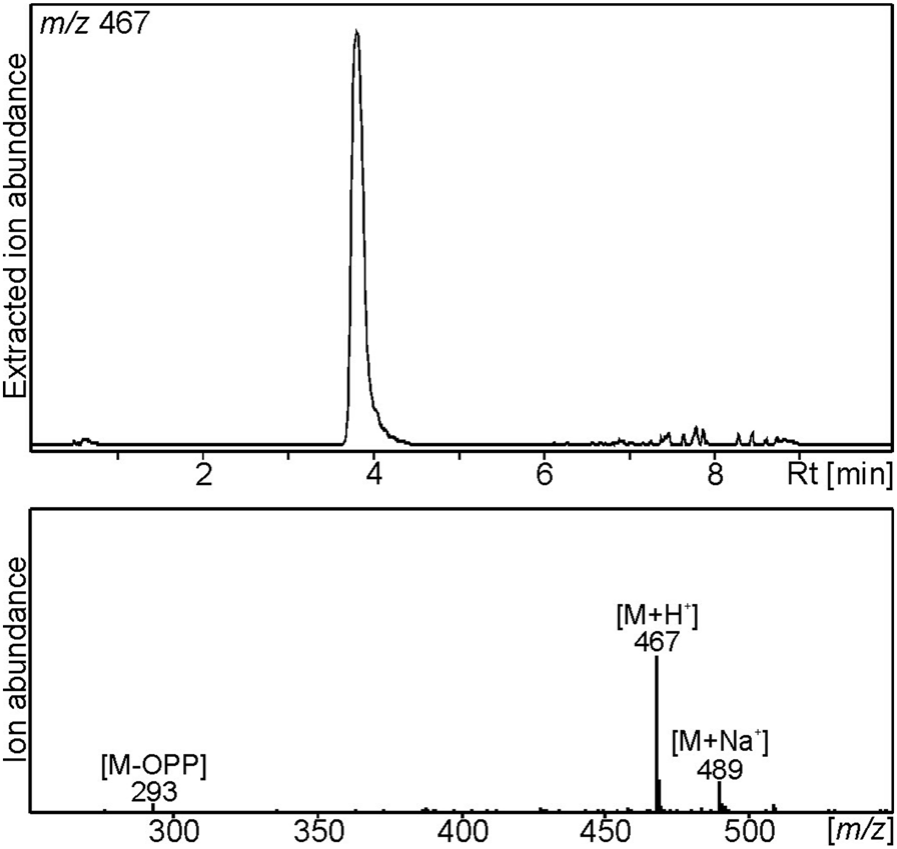
**LC-MS analysis of *Ss*LPPS reaction products.** Normal phase LC-APCI-MS analysis in negative mode was used to directly detect the *Ss*LPPS reaction product from GGPP as substrate without dephosphorylation. Results are depicted as extracted ion chromatogram (EIC) of the parent mass *m*/*z* 467, which is consistent with labda-13-en-8-ol diphosphate (LPP) **3**.

While the FL-cDNA of *Ss*LPPS was directly amplified from calyx cDNA, the obtained FL-cDNAs of *Ss*SS and *Ss*diTPS3 were subjected to codon-optimization for expression of the synthesized genes in *E. coli*. To investigate the catalytic activity of *Ss*LPPS, *Ss*SS and *Ss*diTPS3, N-terminally truncated constructs were generated that lack putative plastidial targeting peptides (Additional file 1: Figure S1). Recombinant proteins were expressed in *E. coli* and Ni^2+^-affinity purified, resulting in soluble proteins of the expected molecular weight of 83 kDa for *Ss*LPPS, 61 kDa for *Ss* SS, and 85 kDa for *Ss*diTPS3. Initial *in vitro* enzyme assays were carried out with GGPP as substrate to test the three enzymes for diTPS activity. For the characterization of *Ss*LPPS, the reaction products were dephosphorylated prior to GC-MS analysis. By comparison to reference mass spectra databases (NIST, Wiley W9N08L), and the product of *Cc*CLS [25], the major product of *Ss*LPPS was identified, after dephosphorylation, as labd-13-en-8,15-diol (labdenediol) (Figure 3 and Additional file 2: Figure S2). Labdenediol, which was absent from the product profile without enzymatic dephosphorylation, is the dephosphorylated form of labda-13-en-8-ol diphosphate (LPP, Figure 1). Additional minor components of the *Ss*LPPS product profile were *epi*-manoyl oxide, manoyl oxide, traces of sclareol, copalol,13(16),14-labdien-8-ol, and an unidentified diterpene compound, with the latter three compounds being of too low abundance to allow unambiguous identification (Figure 3 and Additional file 2: Figure S2). Additional LC-MS analysis on non-dephosphorylated reaction products confirmed LPP as the major product of *Ss*LPPS (Figure 4), which identified *Ss*LPPS as an LPP synthase.

**Figure 5:**
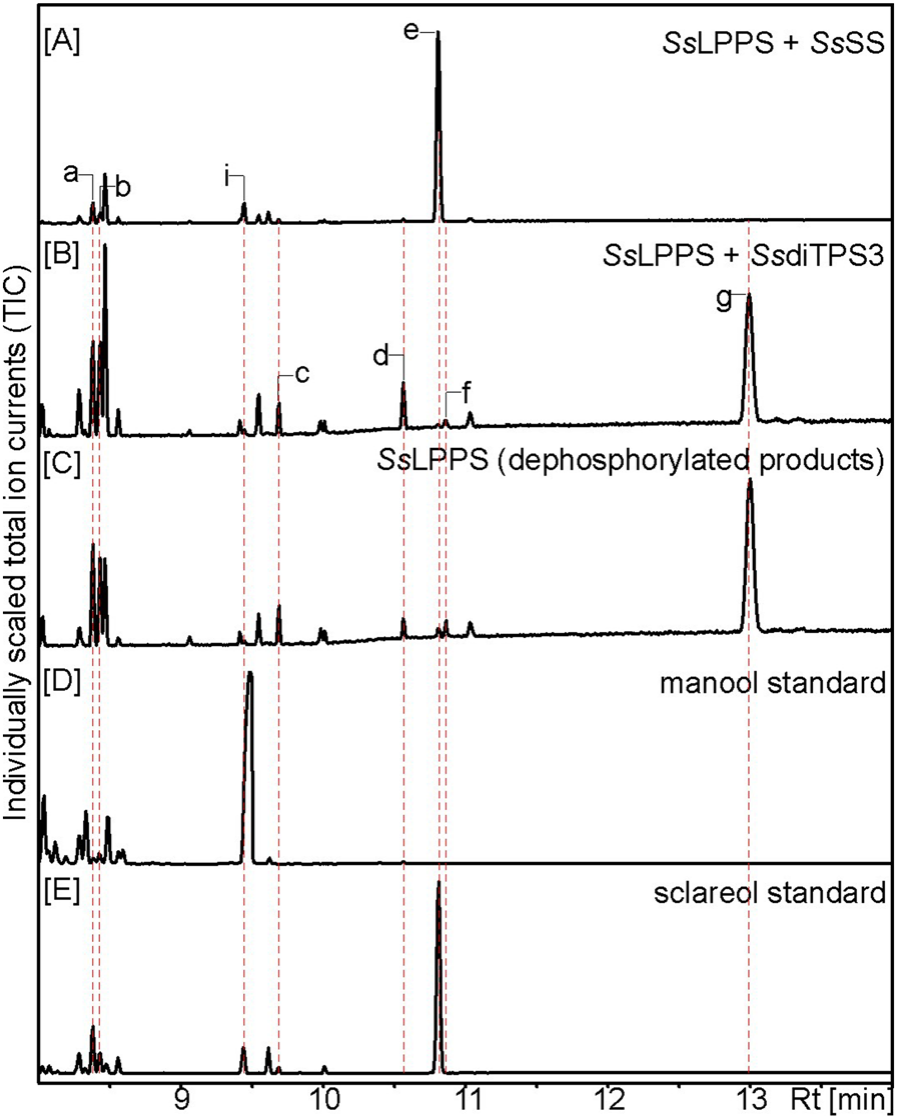
**GC-MS analysis of *Ss*SS and *Ss*diTPS3 reaction products.** Shown are total ion chromatograms (TIC) of reaction products from coupled enzyme assays with equal amounts of *Ss*LPPS and either *Ss*SS or *Ss*diTPS3 using GGPP **1** as a substrate. [**A**] Direct reaction products from coupled assays with *Ss*LPPS and *Ss*SS, [**B**] direct reaction products from coupled assays with *Ss*LPPS and *Ss*diTPS3, [**C**] dephosphorylated reaction products of *Ss*LPPS alone, [**D**] authentic manool standard (GlycoSyn, Gracefield, NZ), and [**E**] authentic sclareol standard (Sigma). GC-MS analysis was performed on an Agilent HP5ms column with electronic impact ionization at 70 eV. Results were confirmed with three independent experiments. Identification of reaction products was achieved by comparison to authentic standards or reference mass spectra from the National Institute of Standards and Technology MS library searches (Wiley W9N08L): peak a, *epi*-manoyl oxide **8**; peak b, manoyl oxide **7**; peak c, putative 13(16),-14-labdien-8-ol; peak d, putative copalol; peak e, sclareol **4**; peak f, unknown compound; peak g, labda-13-en-8,15-diol; peak i, manool **6**.

*Ss*SS and *Ss*diTPS3 were not active with GGPP as substrate. We further tested *Ss*SS and *Ss*diTPS3 in coupled enzyme assays with *Ss*LPPS. In assays with *Ss*LPPS and *Ss*SS, sclareol was the major product with minor amounts of manool, manoyl oxides and *epi*-manoyl oxide as secondary products (Figure 5 and Additional file 2: Figure S2). These products were identified by comparison to the authentic compounds and reference mass spectra. According to these results from *in vitro* enzyme assays, *Ss*SS was identified as a monofunctional class I sclareol synthase, which converts LPP produced by *Ss*LPPS to sclareol, as the second diTPS-catalyzed reaction in sclareol biosynthesis (Figure 1).

Combination of *Ss*LPPS and *Ss*diTPS3 yielded no additional product peaks as compared to *Ss*LPPS alone, suggesting that *Ss*diPTS3 is not able to use LPP as a substrate (Figure 5). Additional coupled assays with *Zea mays ent*-CPS [45] and a protein variant of *Picea abies Pa*LAS producing (9*R*,10*R*)-CPP (i.e. *ent*-CPP) and (9 *S*,10 *S*)-CPP as alternative substrates, respectively, also did not reveal activity of *Ss*diTPS3 with CPP (data not shown).

### Sclareol production in engineered yeast

**Figure 6:**
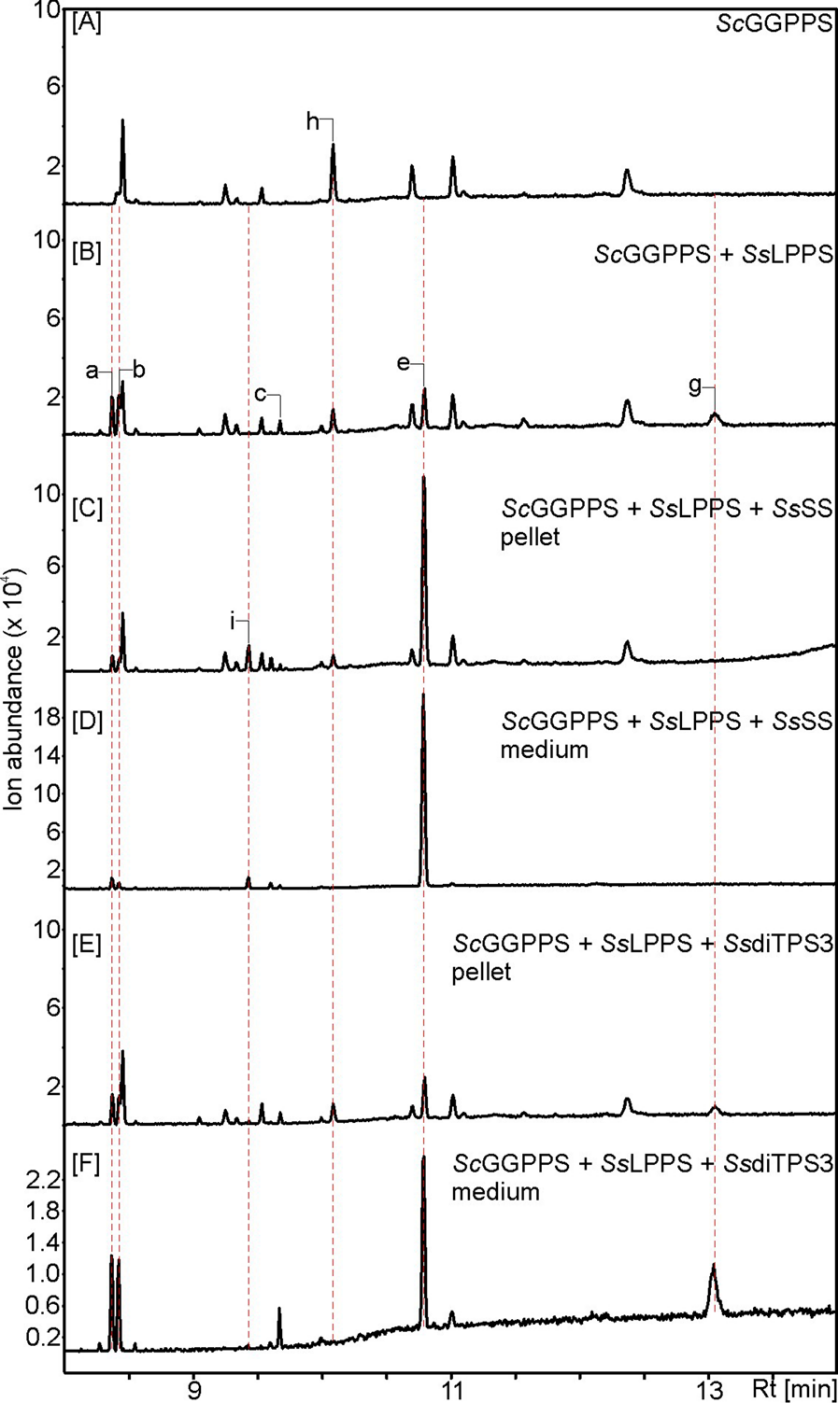
**Production of sclareol in engineered yeast cells.** Shown are GC-MS total ion chromatograms (TIC) of extracts from engineered yeast strains expressing [**A**] yeast GGPP synthase (*Sc*GGPPS), [**B**] *Sc*GGPPS and *Ss*LPPS, [**C-D**] *Sc*GGPPS, *Ss*LPPS and *Ss*SS, and [**E-F**] *Sc*GGPPS, *Ss*LPPS and *Ss*SdiTPS3.Sclareol was detected at similar levels in both the media [**D** &**F**] and harvested yeast cells [**C** &**E**] of each yeast culture, with a lower amount of impurities being observed in the media. GC-MS analysis was performed on an Agilent HP5ms column with electronic impact ionization at 70 eV. Identification of reaction products was achieved by comparison to authentic standards or reference mass spectra from the National Institute of Standards and Technology MS library searches (Wiley W9N08L): peak a, *epi*-manoyl oxide **8**; peak b, manoyl oxide **7**; peak c, putative 13(16),-14-labdien-8-ol; peak d, putative copalol; peak e, sclareol **4**; peak f, unknown compound; peak g, labda-13-en-8,15-diol; peak i, manool **6**.

To substantiate our results of *Ss*LPPS and *Ss*SS functions determined in *in vitro* assays, we used heterologous expression in yeast (*S. cerevisiae*) for additional *in vivo* assays. Metabolically-engineered yeast would also be a suitable biological system for scalable, and potentially industrial, production of sclareol. In a modular engineering approach, yeast cells were co-transformed with a *S. cerevisiae* GGPP synthase (*Sc*GGPPS) [29] and *SsLPPS* alone, or in combination with *SsSS* or *SsdiTPS3*. Yeast strains expressing only *Sc*GGPPS or carrying an empty vector were used as controls. After induction with galactose, both culture media and yeast cell pellets were collected and extracted with pentane. GC-MS analysis of the resulting pentane extracts showed similar results to the *in vitro* enzyme assays described above. Only the combination of *Ss*LPPS and *Ss*SS afforded sclareol as the major product, while expression of *Ss*LPPS alone resulted in only trace amounts of sclareol (Figure 6). Co-expression of *Ss*LPPS with *Ss*SdiTPS3 did not yield any additional product formation compared to expression with *Ss*LPPS or *Sc*GGPPS alone. Even though sclareol yield and purity in the culture media was not quantitatively measured, its accumulation in the medium suggests an active or passive release from the engineered yeast cells. These findings outline a promising basis for developing microbial production systems as an alternative to production in plants, where a clary sage field will produce from 10 to 15 Kg of inflorescence per hectare every other year (biannual plant) with a sclareol content of 1 to 2.5%. Sclareol is then extracted with an organic solvent and purified by a physical process to 95% purity. The overall extraction and purification yield can be up to around 35%.

### Subcellular localization of SsLPPS and SsSS

**Figure 7:**
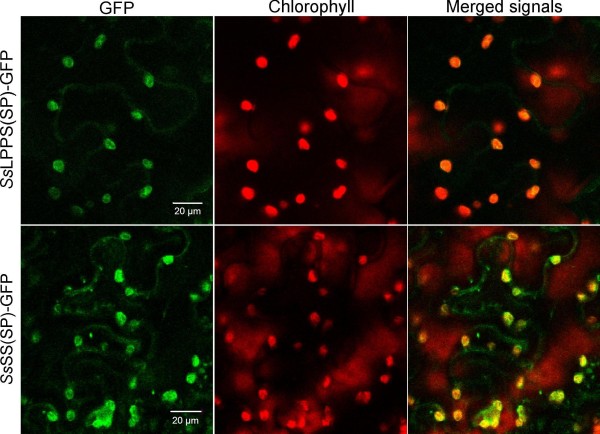
**Subcellular localization of *Ss*LPPS and *Ss*SS.** The first 102 bp of *Ss*LPPS (*Ss*LPPS(SP)-GFP) and the first 99 bp of *Ss*SS (*Ss*SS(SP)-GFP) were fused to a downstream GFP and transiently expressed in tobacco leaves. GFP fluorescence (green) was detected at an emission wavelength of 485–520 nm as compared to chlorophyll autofluorescence (red) at an emission wavelength of 600–700 nm. The column labeled “Merged signals” provides a view of all fluorescent signals obtained for this sample.

The biosynthesis of sclareol is believed to occur in plastids as the substrate GGPP is predominantly derived from the plastidial 2-C-methyl-D-erythritol-4-phosphate (MEP) pathway [40]. To validate this hypothesis individually for *Ss*LPPS and *Ss*SS, we evaluated their subcellular distribution by transient expression of individual green fluorescent protein (GFP)-fusion proteins and confocal laser scanning microscopy. For this purpose, the putative plastidial signal peptides (SP) of *Ss*LPPS and *Ss*SS were fused in frame with the N-terminus of GFP and transiently expressed in *N. benthamiana* leaves. Confocal microscopy two to four days after transformation demonstrated a plastidial localization of the SP-GFP fusions for *Ss*LPPS and *Ss*SS (Figure 7).

## Discussion

Naturally occurring diterpenol metabolites such as manool, *cis-*abienol or sclareol are of high value to the fragrance industry. For example, sclareol is commercially produced from clary sage plantations and used as a primary material in perfume manufacture.

In this study, we demonstrated that sclareol is biosynthesized through a two-step cyclization of GGPP, by two monofunctional diTPSs isolated from clary sage, namely *Ss*LPPS and *Ss*SS. We showed that the introduction of oxygen functionalities in sclareol biosynthesis is catalyzed by diTPSs and does not require activity of, for example, cytochrome P450 monooxygenases.

Similar to a class II diTPS from *C. creticus* (*Cc*CLS) [25], *Ss*LPPS catalyzes the formation of LPP, through a protonation-initiated cyclization of the substrate GGPP, followed by capture of a hydroxyl ion at C-8, as previously suggested. After the recent report of *Cc*CLS [25], *Ss*LPPS is only the second monofunctional class II diTPS reported to facilitate the formation of an oxygen-containing diterpene core structure. The close phylogenetic relationship to other class II diTPSs within the TPS-c family indicates that *Ss*LPPS arose from a CPS gene potentially involved in general gibberellin metabolism via gene duplication and neo-functionalization, resulting in a diTPS for the formation of LPP as the major product in specialized metabolism.

The subsequent *Ss*SS-catalyzed class I reaction proceeds via the ionization of the diphosphate ester of LPP and hydroxylation at C-13, generating the diterpenediol product, sclareol. In contrast to the newly identified monofunctional *Ss*SS from an angiosperm plant species, all of the previously reported diTPSs which catalyze hydroxylations during class I reactions, such as CPS/KS from *P. patens* producing *ent*-16α-hydroxy-kaurene [37], CPS/KSL from *S. moellendorffii* that forms labda-7,13-dien-15-ol [27], and *Pa*LAS recently shown to produce the tertiary diterpenol 13-hydroxy-8(14)-abietene [26], were bifunctional class I/II diTPSs from non-vascular or gymnosperm plants. With the additional hydroxylation at C-13, *Ss*SS adds a novel function to the portfolio of diTPSs that introduce hydroxy functionality to the hydrocarbon backbone of diterpenes and deepens our understanding of the catalytic space of diTPSs that contributes to the remarkable diversity of naturally occurring plant diterpenoids.

Our results support independently the claims of a recent, non-peer-reviewed, patent report [36] of a class II diTPS ([GenBank: AET21247]; 98.9% amino acid identity to *Ss*LPPS) and a class I diTPS ([GenBank: AET21246]; 99.7% identity to *Ss*SS) from *S. sclarea*. The patent also reported LPP and manoyl oxides as the primary products of the class II diTPS and formation of sclareol when class I and class II diTPS were fused. Our data provide, nevertheless, a more complete functional characterization of the substrate and product specificities of these genes.

In our work, GC-MS analysis of reaction products of the combined activities of *Ss*LPPS and *Ss*SS with GGPP as the starting substrate demonstrated the formation of minor amounts of manool in addition to sclareol, which could originate from the conversion of CPP as a side product of the *Ss*LPPS-catalyzed reaction. While sclareol is the most abundant diterpene found in the essential oils of *S. sclarea* with manool as a minor diterpene, other *Salvia* species such as *S. oligophylla*[46] and *S. argentea*[47] exhibit a high abundance of manool but not sclareol. Recent studies in rice and wheat have demonstrated that class I diTPSs can act on substrates of different size and stereochemistry [23,43]. The small quantities of CPP detected in the product profile of *Ss*LPPS as well as the presence of manool as a minor product of the coupled reaction with *Ss*SS suggest that *Ss*SS can convert both (9 *S*,10 *S*)-CPP and LPP to form manool and sclareol, respectively. This hypothesis will be tested in future work. In general, the cloning and functional characterization of *Ss*LPPS and *Ss*SS from *S. sclarea* will enable the discovery of the potentially orthologous diTPS in other *Salvia* species, which will shed light on the molecular processes that determine the selective formation of sclareol *versus* manool as major products in the different species. Such knowledge will be useful for the targeted molecular breeding of *Salvia* species for the fragrance industry.

Subcellular localization studies supported the conclusion that *Ss*LPPS and *Ss*SS are targeted to plastids. This result further suggests that sclareol biosynthesis occurs in the plastids of flower calyces where the corresponding diTPS transcripts were highly abundant [38] and that precursors are most likely derived from the plastidial MEP pathway.

Both *Ss*LPPS and *Ss*SS are phylogenetically related to other diTPSs involved in specialized diterpene metabolism. *Ss*LPPS is closely related to the *Cc*CLS [25], which has the same enzymatic function, and a CPS from *S. miltiorrhiza* involved in tanshinone biosynthesis [24]. *Ss*SS groups with KS-like enzymes and shares almost 60% identity with a miltiradiene synthase from *S. miltiorrhiza*[48]. Interestingly, these two enzymes do not exhibit the common αβγ-domain structure of archetype plant diTPSs [41,44], indicating a loss of the γ-domain in a common ancestor. Such events of domain loss may ultimately have resulted in the evolution of mono- and sesqui-TPSs from ancestral αβ-domain diTPS enzymes [43].

*Ss*diTPS3 appears phylogenetically closer related to *ent*-KS involved in general metabolism. However, neither *in vitro* nor yeast *in vivo* assays revealed diTPS activity with *ent*-CPP, (9 *S*,10 *S*)-CPP, or LPP as a substrate. In addition, *Ss*diTPS3 did not exhibit class II activity as no conversion of GGPP was observed. Lack of diTPS activity of *Ss*diTPS3 may be due to a mutation of the conserved Asn of the NSE/DTE functional motif to His in *Ss*KSL2, since an Asn in this position has previously been shown to be critical for the class I reaction by coordinating the Mg^2+^ cluster in the class I active site [42,49,50].

The two hydroxyl groups in sclareol are responsible for most of the market value of this substance because most of the harvested sclareol is chemically modified to generate high value commercial hemisynthetic products such as Ambrox®. Without such hydroxyl groups, the labdane hydrocarbon backbone would be unreactive. Due to the unique properties of the activities of *Ss*LPPS and *Ss*SS to catalyze distinct position-specific hydroxylation reactions during the cycloisomerization of GGPP via LPP to sclareol, these new enzymes are of great significance for the metabolic engineering of heterologous production systems for sclareol and potentially other oxygenated diterpene bioproducts. It is important to note that the introduction of oxygen functionalities by diTPSs, without requirement for P450 activities, provides a substantial advantage for metabolic engineering of both prokaryotic and eukaryotic host systems, since TPS enzymes are inherently more amenable to expression in a range of heterologous hosts systems than P450 enzymes. Indeed, engineering of *Ss*LPPS and *Ss*SS into yeast provided independent evidence, in addition to *in vitro* assays, for the enzymatic production of sclareol by *Ss*LPPS and *Ss*SS without the requirement of a *S. sclarea* P450 enzyme for oxidation of the diterpene.

The use of engineered microbial platforms for industrial-scale production of high-value terpenes has emerged as a viable approach, especially for the manufacture of pharmaceutical agents, such as artemisinin and taxol [9,48,51,52]. Formation of sclareol through co-expression of *Ss*LPPS and *Ss*SS with GGPPS in yeast shown here, represents a proof-of-concept for future efforts to develop a simple and reliable sclareol production system that is independent of environmental factors in agricultural production. It should be noted that sclareol accumulated in both the cell pellets and the culture media to approximately similar levels, yet with a higher purity in the media, which may allow for efficient extraction of sclareol from fermentation systems. Interestingly, a recent study on the closely related miltiradiene-producing diTPSs from *S. miltiorhizza* demonstrated the interaction of the class II and class I enzymes and further application of these findings allowed the optimization of microbial miltiradiene production through fusion of both proteins, *bona fide* precluding dilution of potentially short-lived intermediates [48]. In the case of sclareol biosynthesis, efficient production was observed when *Ss*LPPS and *Ss*SS were disjoint during *in vitro* and *in vivo* assays. Yet, their uniform subcellular localization supports an interaction of both enzymes and future studies may reveal if such metabolite channelling can be implemented to accelerate sclareol production.

## Conclusions

In conclusion, the new knowledge of diTPSs of sclareol biosynthesis and their successful expression for sclareol formation in yeast provides a robust foundation for the development of a scalable and sustainable production system, applicable in the fragrance industry.

## Methods

### Isolation and cloning of FL-cDNAs

Sequences for putative diTPS transcripts representing members of the TPS-c or TPS-e/f clades were previously identified in the transcriptome resource developed from *S. sclarea* calyces [38]. For PCR amplification of the corresponding cDNAs, total RNA was extracted from *S. sclarea* calyces using the Tri reagent kit (Euromedex, http://www.euromedex.com), and first strand cDNAs were obtained from 2 μg of total RNA using the M-MLV Reverse Transcriptase (Promega, http://www.promega.com). Unique, target-specific oligonucleotide primers were designed using Primer 3 plus (Additional file 3: Table S1). PCRs were carried out with the GoTaq DNA polymerase (Promega) in a final volume of 50 μl including 0.8 μM of each primer, 0.2 mM of each dNTP, and 4 μl of a 5-fold dilution of first strand cDNAs. The reactions were heated for 2 min to 95°C followed by 30 cycles of 95°C for 30 sec, 55°C for 30 sec, 72°C for 75 sec and followed by a final extension at 72°C for 5 min. To obtain FL-cDNAs of the three candidate diTPS genes, 5’ and 3’ RACE-PCR were performed with the Marathon cDNA amplification kit (Clonetech*,*http://www.clontech.com) according to the manufacturer’s instructions. The cDNA template was made from 1 μg of total RNA. Gene-specific primers used are listed in Additional file 4: Table S1. All PCR products were cloned into the pGEM-T Easy vector (Promega).

The EST library that was data mined for diTPS sequences [38] was normalized before sequencing and therefore prevented estimations of *Ss*DTPS transcript abundances. ESTs contigs Salvi_c6071 and Salvi_c2272 had a combined length of 1455 bp and covered 84.3% of the 1725 bp of *Ss*SS. The 454 read FE21XKK02HIAG9 contained a coding sequence of 189 bp which covered only 8.1% of the 2322 bp of *Ss*diTPS3. The combined EST contigs Salvi_c1504, Salvi_c10842, Salvi_c12957 and Salvi_c17648 and the 454 read FE21XKK02JPKR covered 1284 bp of the 2355 bp of the coding sequence of SsLPPS, i.e., 54.4%.

The cDNA sequences described in this paper have been submitted to the GenBank: TM/EBI Data bank with accession numbers: JQ478434 (*Ss*LPPS), JQ478435 (*Ss*SS) and JQ478436 (*Ss*diTPS3).

For protein expression in *Escherichia coli*, the FL-cDNA for *Ss*LPPS was cloned from calyx cDNA. Codon optimized FL-cDNAs for protein expression in *E. coli* of *Ss*SS and *Ss*diTPS3 were synthesized at GeneArt (http://www.geneart.com – sequences in Additional file 4: Figure S3). N-terminal truncations of *Ss*LPPS (Δ65), *Ss*KSL1 (Δ53) and *Ss*KSL2 (Δ29), lacking the predicted putative transit peptides, were established by PCR amplification using the FL constructs as template and cloned into the pET28b(+) expression vector (EMD Biosciences, http://www.emdbiosciences.com) in frame with the N-terminal hexahistidin tag.

For protein expression in yeast, the N-terminal truncated version of *Ss*LPPS was sub-cloned into the first multiple cloning site of the expression vector pESC-Leu (Stratagene, http://www.genomics.agilent.com), resulting in pESC-Leu:*Ss*LPPS. Truncated cDNAs of *SsSS* and *SsdiTPS3* were then individually sub-cloned into the second multiple cloning site of pESC-Leu:*Ss*LPPS, resulting in pESC-Leu:*Ss*LPPS/*Ss*SS and pESC-Leu:*Ss*LPPS/*Ss*diTPS3, respectively. These constructs were individually co-transformed with the previously described plasmid pESC-His:*Sc*GGPPS [29], containing a GGPP synthase from *Saccharomyces cerevisiae*, into the yeast strain BY4741.

### Phylogenetic analysis

Multiple protein sequence alignments were performed using the DIALIGN web server (http://bibiserv.techfak.uni-bielefeld.de/dialign/). Phylogenetic analyses were conducted on the basis of the maximum likelihood algorithm using PhyML 3.0 [53] with four rate substitution categories, LG substitution model, BIONJ starting tree and 100 bootstrap repetitions, and displayed as unrooted tree using treeview32 1.6.6 [54].

### Protein expression and purification

Constructs in the pET28b(+) vector were transformed into *Escherichia coli* BL21DE3-C41 cells [55], and proteins expressed and purified as previously described [56]. Recombinant proteins were then Ni^2+^-affinity purified on a 1 ml HisTrap column (GE Healthcare, http://www.gehealthcare.com) and further desalted against 50 mM HEPES (pH 7.0), 150 mM NaCl, 10% glycerol, 1 mM DTT using PD MiniTrap G-25 columns (GE Healthcare) as previously described [56].

### *In vitro* enzyme assays

Enzymes assays were carried out as described before [56,57] in 50 mM HEPES (pH 7.0), 10 μM MgCl_2_, 5% glycerol, using 20 μg of purified protein (20 μg each for coupled assays) and 20 μM of GGPP (Sigma, http://www.sigmaaldrich.com) as substrate. Reactions were allowed to proceed for 1 h at 30°C with gentle shaking. For the detection of diphosphate intermediates, reaction products were dephosphorylated by incubation with 7 U of calf intestinal alkaline phosphatase (Invitrogen, http://www.invitrogen.com) for 16 h at 37°C. Extraction of reaction products was achieved by vortexing the samples with 500 μl of pentane twice for 20 sec and subsequent centrifugation for 15 min at 1,000 × *g*, 4°C.

### Production of sclareol in engineered yeast cells

pESC-HIS:*Sc*GGPPS was transformed in *S. cerevisiae* (BY4741) in combination with either pESC-LEU:*Ss*LPPS, pESC-LEU:*Ss*LPPS/*Ss*SS or pESC-LEU:*Ss*LPPS/*Ss*diTPS3. Yeast transformation, growth media, and culture conditions were described previously [29,30,58]. Cells were grown in 50 mL of 2% dextrose and Leu/His dropout selective medium up to an OD_600_ of 0.6 to 0.8, at which point yeast cells were transferred to 50 mL of 2% galactose and Leu/His dropout selective medium for 20–24 h. Yeast cells were separated from the medium and 500 μL of medium was extracted with 500 μL of pentane. The harvested cells were extracted twice with 5 mL of pentane, and extracts were concentrated under N_2_ to 500 μL prior to gas chromatographic-mass spectrometric (GC-MS) analysis.

### Gas chromatographic-mass spectrometric (GC-MS) analysis

GC-MS analysis was performed by electronic ionization (EI) at 70 eV after injection of 1 μL of the pentane overlay into an Agilent 6890A GC, 7683B series autosampler (vertical syringe position of 7), combined with a 5973 N Inert XL MS detector at 70 eV. Compound separation was achieved on a SGE SOLGEL-WAX (30 m × 250 μm i.d., 0.25 μm film thickness) column with 1 mL min^-1^ He as carrier gas, using the following GC temperature program: 40°C for 2 min, ramp at a rate of 25°C min^-1^ to 250°C, hold 5 min, pulsed splitless injector held at 250°C. Compounds were identified by comparison to authentic standards and reference mass spectra databases (Wiley W9N08L and the National Institute of Standards and Technology MS library searches). The authentic sclareol standard was purchased from Sigma.

### Liquid chromatographic (LC)-MS Analysis

LC-MS analysis was conducted on an Agilent 1100 LC/MSD Trap XCT Plus mass spectrometer. Compound separation was achieved on an Agilent Zorbax SB-C18 column (50 mm × 4.6 mm ID, 1.8 μm) with isocratic elution of CH_3_CN/NH_4_HCO_3_ (5%/95%) pH 7.95 as mobile phase at 35°C and a flow rate of 1.2 ml min^-1^. Mass spectrometric analysis was performed after electrospray ionization (ESI) in negative mode with a scan range of *m*/*z* 100–600.

### Subcellular localization of SsLPPS and SsSS

The GFP fusion constructs for transient expression in *Nicotiana benthamiana* were prepared as follows. The predicted N-terminal signal peptides of *Ss*LPPS[*Ss*LPPS(SP); 1–102 bp] and *Ss*SS [*Ss*SS(SP); 1–99 bp] were amplified with gene-specific primers and cloned into pENTR/D-TOPO (Invitrogen). The resulting clones were transferred by Gateway LR reactions as recommended by the manufacturer (Invitrogen) into the destination vector pMDC83, in-frame with a C-terminal green fluorescent protein (GFP).

*N. benthamiana* seeds were disinfected with chlorine fumes for 6 h and placed on Murashige and Skoog basal salt medium (Sigma) containing 0.7% agar. After incubation for 2 weeks at 24°C, seedlings were transferred to soil and the plants were grown in a chamber with a 16 h photoperiod and a temperature range of 22°C (night time low) to 25°C (day time high). Plants that were 4 to 6 weeks old were used for transformation.

Each expression vector was introduced into *Agrobacterium tumefaciens* strain C58 (pMP90) by chemical transformation as reported elsewhere [59]. Plants were transfected with the fluorescent constructs as described previously [60]. Five mL of LB overnight *A. tumefaciens* cultures containing antibiotics were pelleted for 15 min at 4,000 *g* and 22°C, washed one time and resuspended in sterilized distilled water to an OD_600_ of 0.5 before being infiltrated into the abaxial side of the leaf using a 1 mL syringe (without needle) and gentle pressure. To avoid post-transcriptional gene silencing, *A. tumefaciens* strains carrying the constructs of interest were co-infiltrated with another strain transformed with a binary vector for expression of the viral p19 silencing suppressor protein [61].

Two to four days post-infiltration leaf discs were excised and mounted between slides and coverslips for observation by confocal microscopy. Cell imaging was performed using an Olympus FV-1000 confocal microscope coupled to an Olympus BX61Wimicroscope stand. Leaf samples were gently squeezed between cover and slide glass with a drop of distilled water. Images were recorded using an Olympus UPLFLN 40X objective lens. Excitation/emission wavelengths were 473 nm/485-520 nm for GFP, and 561 nm/600-700 nm for intrinsic fluorescence of the chloroplasts. The images shown in the results are single focal sections acquired using the FluoView Olympus software and directly analyzed with ImageJ [62].

## Abbreviations

diTPS: Diterpene synthase; *Ss*LPPS: Labd-13-en-8-ol diphosphate synthase from *Salvia sclarea*; *Ss*SS: Sclareol synthase from *Salvia sclarea*; *Ss*TPS3: Diterpene synthase 3 from *Salvia sclarea*; GGPP: Geranylgeranyl diphosphate; LPP: Labda-13-en-8-ol diphosphate; CPP: Copalyl diphosphate; CPS/KS: Copalyl diphosphate / kaurene synthase; FL: Full-length; GC-MS: Gas chromatography–mass spectrometry; SP: Signal peptide.

## Authors’ contributions

ACa and PZ conducted the recombinant protein purification, the *in vitro* enzyme assays, the engineering of yeast cells, the tobacco transient expression assays for the subcellular localization of the three diTPS and the phylogenetic analyses. SL and NV isolated and cloned the full-length cDNAs of the three diTPS characterized in this article. SL also contributed to *Ss*LPPS recombinant protein purification and enzyme assay. ACo and J-LM helped in the setting-up of the tobacco transient expression assays. JB and LL designed the study and contributed to the data analysis. LL conceived of the study. ACa, PZ, JB and LL wrote the manuscript with contributions from the coauthors. All partners have read and approved the final manuscript.

## Supplementary Material

Additional file 1 **Figure S1.** Protein sequence alignments. Amino acid sequence alignments of *Ss*LPPS [A] and *Ss*SS and *Ss*diTPS3 [B] were generated in CLC bio as compared to representative class II and class I diterpene synthases. Grey shading indicates strictly conserved residues. The catalytically relevant aspartate-rich motifs (i.e., DxDD, DDxxD, NDxxTxxxE) are highlighted and predicted plastidial transit peptides are underlined. N-terminal truncations for expression of recombinant proteins in *E.coli* and yeast and protein fragments used for subcellular localization studies are marked as red and green arrows, respectively. Abbreviations: *Ss*LPPS, *Salvia sclarea* labda-13-en-8-ol diphosphate synthase [GenBank: JQ478434]; *Nt*CPSL, *Nicotiana tabacum* 8-hydroxy copalyl diphosphate synthase [GenBank: CCD33018]; *Cc*CLS, *Cistus creticus* copal-8-ol synthase [GenBank: ADJ93862]; *Sm*CPSL, *Salvia miltiorhizza* copalyl diphosphate synthase-like [GenBank: EU003997]; *At*CPSL, *Arabidopsis thaliana ent-*copalyl diphosphate synthase [GenBank: AAA53632]; *Ss*SS, *S. sclarea* sclareol synthase [GenBank: JQ478435]; *Ss*diTPS3, *S. sclarea* diterpene synthase-3[GenBank: JQ478436]; *Sm*KSL, *S. miltiorhizza* kaurene synthase-like [GenBank: EF635966]; *At*KS, *A. thaliana ent-*kaurene synthase [GenBank: AF034774]; *Sr*KS, *Stevia rebaudiana* kaurene synthase [GenBank: AAD34295]; *Nt*KSL, *N. tabacum* kaurene synthase-like [GenBank: CCD33019]. Click here for file

Additional file 2 **Figure S2.** Mass spectra of assay products as compared to reference spectra of authentic standards and relevant databases. Illustrated are characteristic mass spectra of enzymatic reaction products as compared to authentic standards or reference mass spectra from the National Institute of Standards and Technology MS library searches (Wiley W9N08L): peak a, 13-*epi*-manoyl oxide 8; peak b, manoyl oxide 7; peak c, putative 13(16)-14-labdien-8-ol; peak d, putative copalol; peak e, sclareol 4; peak f, unknown compound; peak g, labda-13-en-8,15-diol; peak i, manool 6. Click here for file

Additional file 3 **Table S1.** Oligonucleotides used for the amplification of cDNA sequences. Click here for file

Additional file 4 **Figure S3.** Codon optimized sequences of *Ss*SS and *Ss*diTPS3. Codon optimized sequences of *Ss*SS and *Ss*diTPS3 that have been used for *Escherichia coli* and yeast-based heterologous protein expressions are shown in FASTA format. Click here for file
