# Supplementary figures and images for: Discovery and functional characterization of two diterpene synthases for sclareol biosynthesis in Salvia sclarea (L.) and their relevance for perfume manufacture
Source: BMC Plant Biol. 2012 Jul 26;12:119. doi: 10.1186/1471-2229-12-119 (PMC3520730; doi:10.1186/1471-2229-12-119)

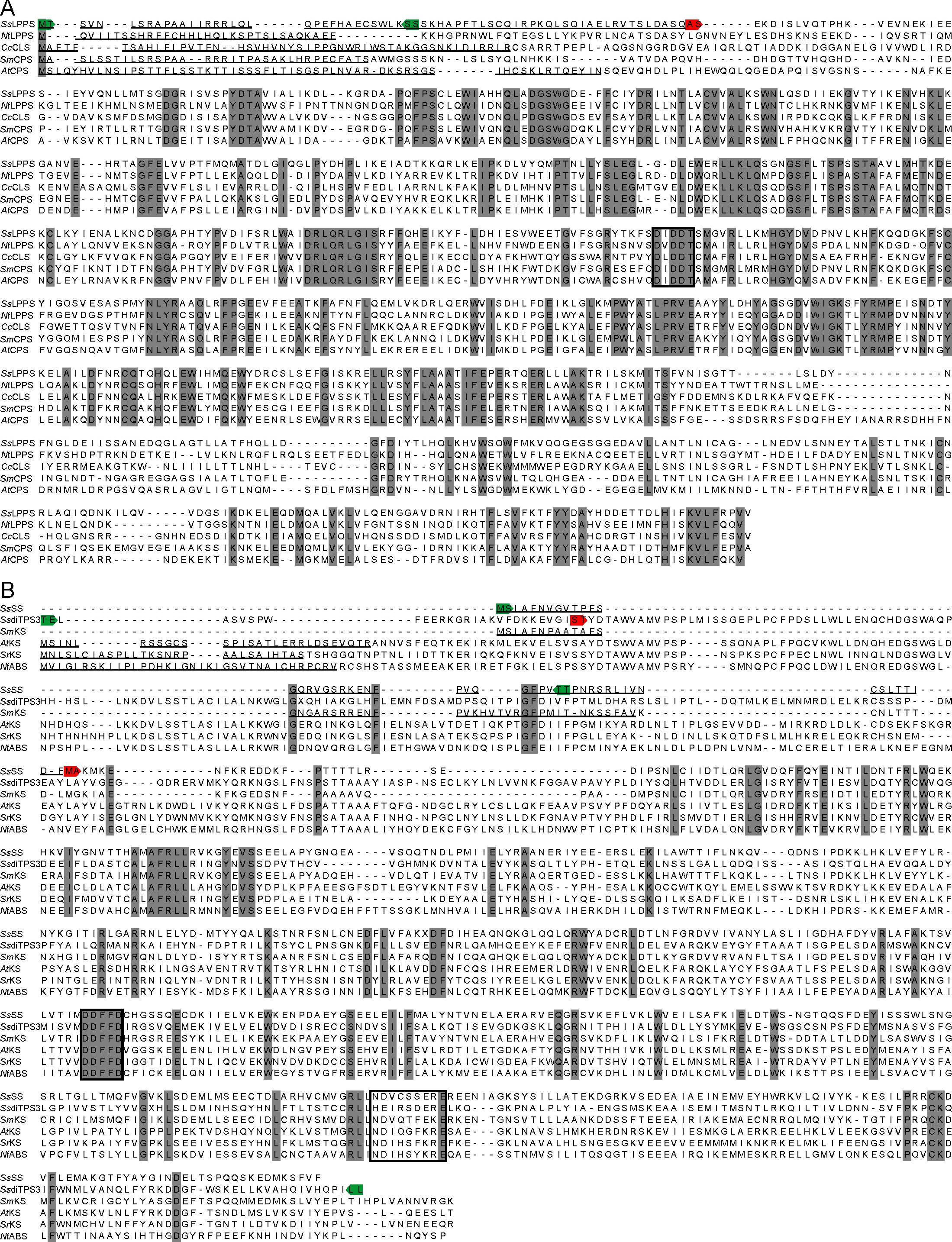

Supplement: Additional file 1 — Figure S1. Protein sequence alignments. Amino acid sequence alignments of SsLPPS [A] and SsSS and SsdiTPS3 [B] were generated in CLC bio as compared to representative class II and class I diterpene synthases. Grey shading indicates strictly conserved residues. The catalytically relevant aspartate-rich motifs (i.e., DxDD, DDxxD, NDxxTxxxE) are highlighted and predicted plastidial transit peptides are underlined. N-terminal truncations for expression of recombinant proteins in E.coli and yeast and protein fragments used for subcellular localization studies are marked as red and green arrows, respectively. Abbreviations: SsLPPS, Salvia sclarea labda-13-en-8-ol diphosphate synthase [GenBank: JQ478434]; NtCPSL, Nicotiana tabacum 8-hydroxy copalyl diphosphate synthase [GenBank: CCD33018]; CcCLS, Cistus creticus copal-8-ol synthase [GenBank: ADJ93862]; SmCPSL, Salvia miltiorhizza copalyl diphosphate synthase-like [GenBank: EU003997]; AtCPSL, Arabidopsis thaliana ent-copalyl diphosphate synthase [GenBank: AAA53632]; SsSS, S. sclarea sclareol synthase [GenBank: JQ478435]; SsdiTPS3, S. sclarea diterpene synthase-3[GenBank: JQ478436]; SmKSL, S. miltiorhizza kaurene synthase-like [GenBank: EF635966]; AtKS, A. thaliana ent-kaurene synthase [GenBank: AF034774]; SrKS, Stevia rebaudiana kaurene synthase [GenBank: AAD34295]; NtKSL, N. tabacum kaurene synthase-like [GenBank: CCD33019]. [file 1471-2229-12-119-S1.jpeg]

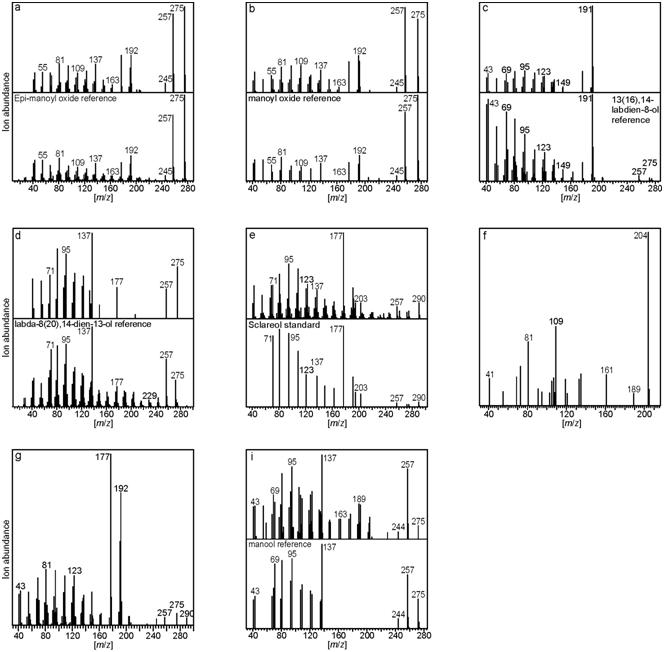

Supplement: Additional file 2 — Figure S2. Mass spectra of assay products as compared to reference spectra of authentic standards and relevant databases. Illustrated are characteristic mass spectra of enzymatic reaction products as compared to authentic standards or reference mass spectra from the National Institute of Standards and Technology MS library searches (Wiley W9N08L): peak a, 13-epi-manoyl oxide 8; peak b, manoyl oxide 7; peak c, putative 13(16)-14-labdien-8-ol; peak d, putative copalol; peak e, sclareol 4; peak f, unknown compound; peak g, labda-13-en-8,15-diol; peak i, manool 6. [file 1471-2229-12-119-S2.jpeg]
