# Supplementary material for: Discovery and functional characterization of two diterpene synthases for sclareol biosynthesis in Salvia sclarea (L.) and their relevance for perfume manufacture
Source: BMC Plant Biol. 2012 Jul 26;12:119. doi: 10.1186/1471-2229-12-119 (PMC3520730; doi:10.1186/1471-2229-12-119)
Supplement: Additional file 3 — Table S1. Oligonucleotides used for the amplification of cDNA sequences. [file 1471-2229-12-119-S3.docx]

>SsSS

ATGTCACTGGCATTTAATGTTGGTGTTACCCCGTTTAGCGGTCAGCGTGTTGGTAGCCGTAAAGAAAACTTTCCGGTTCAGGGTTTTCCGGTTACCACCCCGAATCGTAGCCGTCTGATTGTTAATTGTAGCCTGACCACCATTGATTTTATGGCCAAAATGAAAGAAAACTTCAAACGCGAAGATGATAAATTTCCGACCACCACCACCCTGCGTAGCGAAGATATTCCGAGCAATCTGTGTATTATTGATACCCTGCAGCGTCTGGGTGTTGATCAGTTTTTTCAGTATGAAATTAATACCATTCTGGATAATACCTTTCGCCTGTGGCAGGAAAAACACAAAGTTATTTATGGCAATGTGACCACCCATGCAATGGCATTTCGTCTGCTGCGTGTTAAAGGTTATGAAGTTAGCAGCGAAGAACTGGCACCGTATGGTAATCAGGAAGCAGTTAGCCAGCAGACCAATGATCTGCCGATGATTATTGAACTGTATCGTGCAGCCAATGAACGCATTTATGAAGAAGAACGTAGCCTGGAAAAAATTCTGGCATGGACCACCATTTTTCTGAATAAACAGGTGCAGGATAATAGCATTCCGGATAAAAAACTGCATAAACTGGTGGAATTTTATCTGCGCAATTATAAAGGCATTACCATTCGTCTGGGTGCACGTCGTAATCTGGAACTGTATGATATGACCTATTATCAGGCACTGAAAAGCACCAATCGCTTTAGCAATCTGTGCAATGAAGATTTTCTGGTGTTTGCCAAACAGGATTTTGATATTCATGAAGCCCAGAATCAGAAAGGTTTACAGCAGCTGCAGCGTTGGTATGCAGATTGTCGTCTGGATACCCTGAATTTTGGTCGTGATGTTGTTATTGTGGCCAATTATCTGGCCAGCCTGATTATTGGTGATCATGCATTTGATTATGTGCGTCTGGCATTTGCAAAAACCAGCGTTCTGGTTACCATTATGGATGATTTTTTTGATTGCCATGGCAGCAGCCAGGAATGCGATAAAATCATTGAACTGGTGAAAGAATGGAAAGAAAATCCGGATGCAGAATATGGTTCCGAAGAACTGGAAATTCTGTTTATGGCCCTGTATAATACCGTTAATGAACTGGCAGAACGTGCACGTGTTGAACAGGGTCGTAGCGTTAAAGAATTTCTGGTTAAACTGTGGGTGGAAATTCTGAGCGCCTTTAAAATTGAACTGGATACCTGGTCAAATGGCACCCAGCAGAGCTTTGATGAATATATTAGCAGCAGCTGGCTGAGCAATGGTAGTCGTCTGACCGGTCTGCTGACCATGCAGTTTGTTGGTGTTAAACTGAGTGATGAAATGCTGATGAGCGAAGAATGTACCGATCTGGCACGTCATGTTTGTATGGTTGGTCGTCTGCTGAATGATGTTTGTAGCAGCGAACGTGAACGCGAAGAAAATATTGCAGGTAAAAGCTATAGCATTCTGCTGGCAACCGAAAAAGATGGTCGTAAAGTTAGCGAAGATGAAGCAATTGCCGAAATTAATGAAATGGTGGAATATCATTGGCGCAAAGTTCTGCAGATTGTGTATAAAAAAGAAAGCATTCTGCCGCGTCGTTGCAAAGATGTTTTTCTGGAAATGGCAAAAGGCACCTTTTATGCCTATGGCATTAATGATGAACTGACCAGTCCGCAGCAGAGCAAAGAAGATATGAAATCCTTTGTGTTTTAA

>SsdiTPS3

ATGATTAGCCTGATGCTGAGCAGCAGCAGCCCGTTTCGTAGCAGTCCGTTTAGCCATGCAAGCAGCGCAGCACTGGATCGTCTGCCTCGTGCAACCAAACTGACCACCGAACTGGCAAGCGTTAGCCCGTGGTTTGAAGAACGTAAAGGTCGTATTGCCAAAGTGTTTGATAAAAAAGAAGTGGGCATTAGCACCTATGATACCGCATGGGTTGCAATGGTTCCGAGTCCGCTGATGATTAGCTCAGGTGAACCGCTGCCGTGTTTTCCGGATAGCCTGCTGTGGCTGCTGGAAAATCAGTGTCATGATGGTAGCTGGGCACAGCCGCATCATCATAGCCTGCTGAATAAAGATGTTCTGAGCAGCACCCTGGCATGTATTCTGGCACTGAATAAATGGGGTCTGGGTGATCAGCATATTGCAAAAGGTCTGCATTTTCTGGAAATGAATTTTGATAGCGCAATGGATCCGAGCCAGATTACCCCGATTGGTTTTGATATTGTTTTTCCGACCATGCTGGATCATGCACGTAGCCTGAGCCTGATTCCGACCCTGGATCAGACCATGCTGAAAGAACTGATGAATATGCGTGATCTGGAACTGAAACGTTGTAGCAGCAGTCCGGATATGGAAGCATATCTGGCCTATGTTGGTGAAGGTCAGGATCGTGAACGTGTTATGAAATATCAGCGCAAAAATGGCAGCCTGTTTAATAGCCCGAGCACCACCGCAGCAGCATATATTGCAAGCCCGAATAGCGAATGTCTGAAATATCTGAATCTGGTGGTGAATAAATTTGGTGGTGCAGTTCCGGCAGTTTATCCGCTGGATATTTATAGCCAGCTGCATACCGTTGATGATCTGGAACGTCTGGGTATTAGCCGTTATTTTGTGACCGAAATTGAAAGCGTTCTGGATCAGACATATCGTTGTTGGGTTCAGGGTGATGAAGAAATTTTTCTGGATGCAAGCACCTGTGCACTGGCATTTCGTCTGCTGCGTATTAATGGTTATAATGTTAGCAGCGATCCGGTTACCCATTGTGTTGTTGGTCACATGAACAAAGATGTGAATACCGCACTGGAAGTGTATAAAGCAAGCCAGCTGACCCTGTATCCGCATGAAACCCAGCTGGAAAAACTGAATAGCAGCCTGGGTGCACTGCTGCAGGATCAGATTAGCAGCGCAAGCATTCAGAGCACCCAGCTGCATGCAGAAGTTCAGCAGGCTCTGGATTATCCGTTTTATGCAATTCTGCAGCGTATGGCAAATCGTAAAGCCATTGAACATTATAATTTTGATCCGACCCGCATTCTGAAAACCAGCTATTGTCTGCCGAATAGCGGCAATAAAGATTTTCTGCTGCTGAGCGTGGAAGATTTTAATCGTCTGCAGGCAATGCATCAGGAAGAATATAAAGAATTTGAACGCTGGTTTGTGGAAAATCGTCTGGATGAACTGGAAGTTGCACGTCAGAAAGTGGAATATGGTTATTTTACCGCAGCAGCCACCATTAGCGGTCCGGAACTGAGTGATGCACGTATGAGCTGGGCAAAAAATTGTGTTATGATTAGCGTTATGGATGATTTTTTTGATATTCGTGGCAGCGTGCAGGAAATGGAAAAAATTGTTGAACTGGTGGAACTGTGGGATGTTGATATTAGCCGTGAATGTTGCAGCAATGATGTGAGCATTATTTTTAGCGCACTGAAACAGACCATTAGCGAAGTTGGTGATAAAGGTAGCAAACTGCAGGGTCGTAATATTACACCGCATATTATTGCACTGTGGCTGGATCTGCTGTATAGCTATATGAAAGAAGTCGAATGGTCAGGTAGCTGTAGCAATCCGAGCTTTGATGAATATATGAGCAATGCCAGCGTGTCTTTTGGTCTGGGTCCGATTGTTGTTAGCACCCTGTATGTTGTTGGTCCGCATCTGAGCCTGGATATGATCAATCATAGCCAGTATCATAACCTGTTTACCCTGACCAGCACCTGTTGTCGTCTGCTGCATGAAATTCGTAGTGATGAACGTGAACTGAAACAGGGTAAACCGAATGCACTGCCGCTGTATATTGCAGAAAATGGTAGCATGAGCAAAGAAGCAGCAATTAGCGAAATGATTACCATGAGCAATACCCTGCGTAAACAAATTCTGACCATTGTGCTGGATAATAATTCCGTTTTTCCGAAACCGTGCAAACAAATTTTTTGGAATATGCTGGTTGCCAATCAGCTGTTTTATCGTAAAGATGATGGCTTTTGGAGCAAAGAACTGCTGAAAGTTGCCCATCAGATTGTTCATCAGCCGATTCTGCTGTAA
