# Supplementary material for: Discovery and functional characterization of two diterpene synthases for sclareol biosynthesis in Salvia sclarea (L.) and their relevance for perfume manufacture
Source: BMC Plant Biol. 2012 Jul 26;12:119. doi: 10.1186/1471-2229-12-119 (PMC3520730; doi:10.1186/1471-2229-12-119)
Supplement: Additional file 4 — Figure S3. Codon optimized sequences of SsSS and SsdiTPS3. Codon optimized sequences of SsSS and SsdiTPS3 that have been used for Escherichia coli and yeast-based heterologous protein expressions are shown in FASTA format. [file 1471-2229-12-119-S4.docx]

| Experiment | Experiment details | Primer ID | Primer sequence (5'-> 3') |
| --- | --- | --- | --- |
| Amplification between unique sequences | Amplicon 1 (*Ss*SS) | c6071-F | CCATAAGATTGGGAGCTAGACG |
|  |  | c2272-R | TTCTTCCATCTTTCTCAGTTGC |
|  | Amplicon 2 (*Ss*LPPS) | JPKR3-F | AGGAGTGACGTACATCAAGG |
|  |  | c17648-R | GTTTGGCATCTGTTGAAATCC |
|  | Amplicon 3 (*Ss*LPPS) | c17648-F | GGTGATGTATGGATTGGCAAG |
|  |  | c1504-R | CCTGCATATCCTGTTCTAGCTC |
| RACE PCR | *Ss*SS 5' RACE | *Ss*KSL1-R | TCGGCAGGTCATTTGTTTGCTGGCTAAC |
|  |  | *Ss*KSL1-Nested-R | TGATCGACCCCCAACCTTTGAA |
|  | *Ss*SS 3' RACE | *Ss*KSL1-F | GGCTAGGCATGTCTGTATGGTCGGC |
|  |  | *Ss*KSL1-Nested-F | AGTTCTGAGAGGGAGCGCGAGGAA |
|  | *Ss*diTPS3 5' RACE | *Ss*KSL2-R | TCTGAAGGCTAAGGCACAAGTGGAGGCG |
|  |  | *Ss*KSL2-Nested-R | TCTCTTCGTCGCCCTGCACC |
|  | *Ss*diTPS3 3' RACE | *Ss*KSL2-F | ATTAGATGTTGGGTGCAGGGCGACGAAG |
|  |  | *Ss*KSL2-Nested-F | CGCCTCCACTTGTGCCTTAGCC |
|  | *Ss*LPPS 5' RACE | *Ss*CPSL-R | GGTGGACGACGGCGAAGTGAGGAAGGAG |
|  |  | *Ss*CPSL-Nested-R | CCCACTCCAAATCTCCTAACCCTTC |
| *Ss*LPPS full length CDS amplification |  | *Ss*CPSL-FL-F | ATGACTTCTGTAAATTTGAGCAGAG |
|  |  | *Ss*CPSL-FL-R | TCATACAACCGGTCGAAAGAGTAC |
